# Diaper-embedded urine test device for the screening of urinary tract infections in children: a cohort study

**DOI:** 10.1186/s12887-020-02277-5

**Published:** 2020-08-11

**Authors:** Niko Paalanne, Lotta Wikstedt, Tytti Pokka, Jarmo Salo, Matti Uhari, Marjo Renko, Terhi Tapiainen

**Affiliations:** 1 Department of Pediatrics and Adolescent Medicine, Oulu University Hospital, Oulu, Finland; 2 PEDEGO Research Unit and Medical Research Centre, University of Oulu, Oulu, Finland; 3 Department of Pediatrics, University of Eastern Finland and Kuopio University Hospital, Kuopio, Finland; 4 Biocenter Oulu, University of Oulu, Oulu, Finland

**Keywords:** Urinary tract infection, Screening method, Urine test sensitivity, Urine dipstick, Urinary analysis

## Abstract

**Background:**

There is a need for an easy and sensitive method for screening of urinary tract infections in young children. We set out to test whether a novel diaper-embedded urine test device is feasible and reliable in screening for urinary tract infections.

**Methods:**

This prospective cohort study consisted of young children examined due to a suspected acute urinary tract infection at the Pediatric Emergency Department of the Oulu University Hospital, Finland. We analyzed the same urine samples using three different methods: 1) a diaper-embedded test device applied to the urine pad within the diaper, 2) a urine sample aspirated from the urine pad for the conventional point-of-care dipstick test, and 3) a urine sample aspirated from the urine pad and analyzed in the laboratory with an automated urine chemistry analyzer. The gold standard for confirming urinary tract infection was quantitative bacterial culture.

**Results:**

Urine samples were available from 565 children. Bacterial culture confirmed urinary tract infection in 143 children. Sensitivity of the positive leukocyte screening of the diaper-embedded urine test device was 93.1% (95% CI: 87.4–96.8) and that of the point-of-care urine dipstick analysis was 95.4% (90.3–98.3) in those with both tests results available (*n* = 528). The sensitivity of the positive leukocyte test of the diaper-embedded test device was 91.4% (85.4–95.5) and that of the automated analysis was 88.5% (82.0–93.3) in those with both tests available (*n* = 547). The time to the test result after urination was immediate for the diaper-embedded test, 1–5 min for point-of-care dipstick, and 30–60 min for laboratory-based automated urine chemistry analyzer.

**Conclusions:**

In this prospective study, the diaper-embedded urine test device was an easy and sensitive screening method for UTIs in young children. The main clinical benefit of the diaper-embedded urine test device was that the screening test result was available immediately after urination.

## Background

Urinary tract infections (UTIs) account for 5–14% of pediatric emergency department visits annually [1]. Non-invasive methods are often used for urine sample collection [2]. Novel clean catch methods have been presented to ease the procedure in young children [3, 4]. Alternatively, the aspiration of urine from urine collection pads inserted within diapers has perceived to be convenient method to collect urine samples [2, 5, 6]. Suprapubic aspiration or catheter sample are recommended after a positive screening test result [7]. A positive, quantitative urine culture is the gold standard for UTI diagnosis. Urine culture, however, cannot provide immediate screening results.

An ideal urine sample screening method is sensitive, fast, and noninvasive. For UTI screening, dipstick urine analysis is an inexpensive and widely used method reported to perform well in children [8, 9]. Automated analyzers have been proved to perform well in UTI screening [10, 11], but they are not used as point-of-care tests. Recently, diaper-embedded test devices have become commercially available for UTI screening [12]. However, there are no studies evaluating the diagnostic accuracy of the diaper-embedded test device methods in screening for UTI in acutely ill infants and young children.

We set out to test whether a diaper-embedded urine test device is feasible and sensitive in screening for UTIs in young children in a large prospective cohort study at a pediatric emergency department.

## Methods

### Study design and population

The population of this prospective study consisted of young children examined due to a suspected acute UTI at the Pediatric Emergency Department in the Department of Pediatrics and Adolescent Medicine, Oulu University Hospital, Oulu, Finland. We recruited the children between June 1, 2013 and August 31, 2017. The Ethics Committee of the Northern Ostrobothnia Hospital District at Oulu University Hospital, Oulu, Finland evaluated and approved the study plan (decision number EETTMK 51/2013). Only children whose families gave their written informed consent were enrolled in the study.

**Fig. 1:**
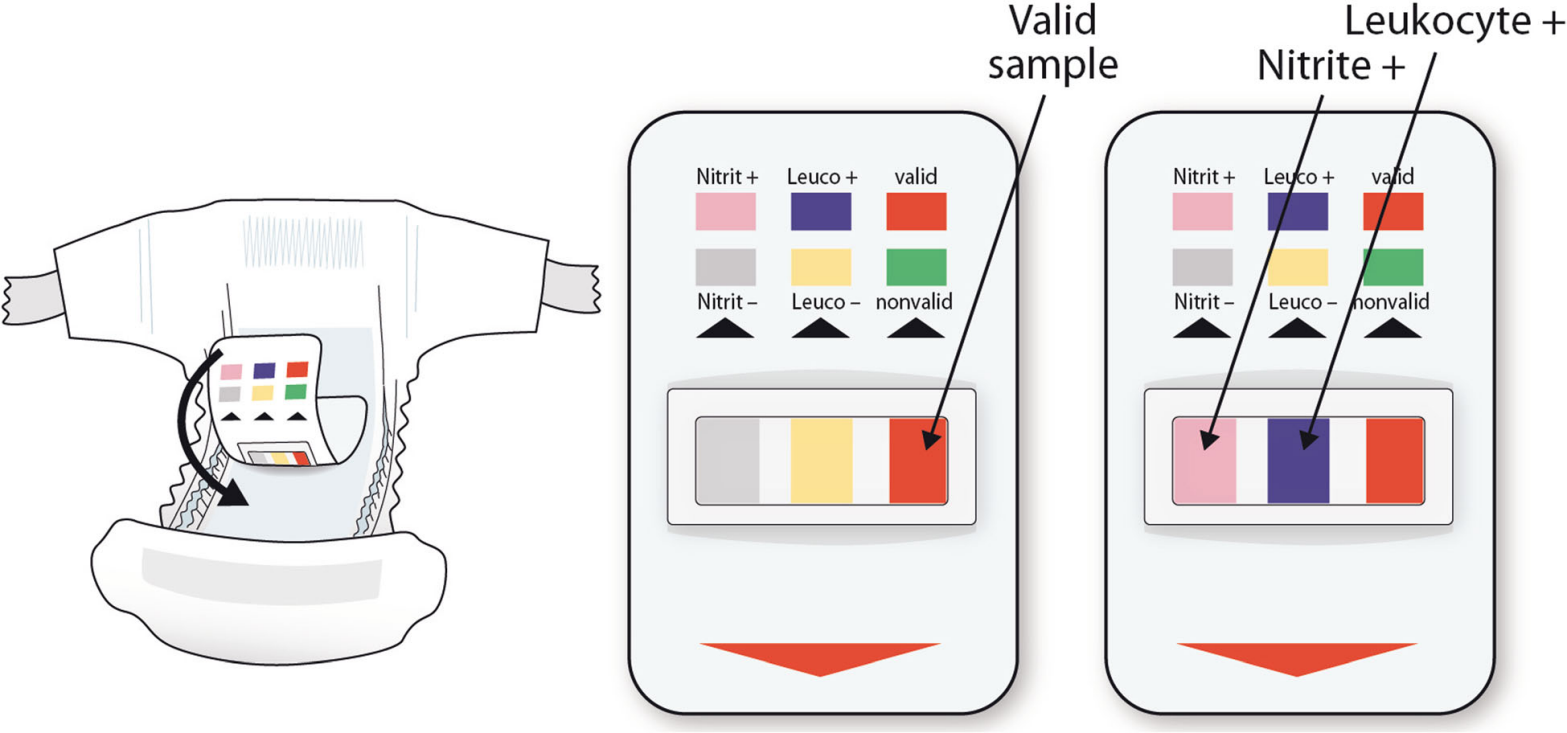
Diaper-embedded urine test device was placed on the urine pad within the diaper. The diaper-embedded urine screening test result was readable immediately after urination. The urine sample was aspirated from the urine pad, placed under the device, for other screening analyses and bacterial culture

We offered the participation to the families whose children wore diapers and were suspected to have a UTI. Trained pediatric nurses explained the study to families, obtained the written informed consent, and placed the diaper-embedded urine test device on the urine pad within the children’s diapers (Fig. 1). The nurses checked the diaper-embedded test device every 30 min. Childrens’ parents reported the easiness of sample collection using a visual analog scale from 0 to 90 mm, zero indicating maximal easiness and 90 indicating maximal difficulty. Any adverse events reported by nurses or parents were collected. The nurses estimated time required for receiving the results after urination We interviewed the nurses concerning easiness of use for different methods.

At least one urine collection pad sample was collected from every child. Method of additional sample collection was selected by treating physicians. If suprapubic aspiration was not performed or clean catch urine was not successfully collected, the second sample was collected with urine collection pad. The method of sample collection was recorded by the nurses as age and gender of the children was collected from electronic patient record (Table 1) Results of the urine dipstick tests and diaper embedded tests were recorded by the nurses. Results of the automated urine analyses were collected by the researchers from electronic patient records.

**Table 1:** Study population of 565 infants and children with a suspected urinary tract infection

Characteristic	Population n (%)		
	Girls	Boys	All
Total	312 (55.2)	253 (44.8)	
Age (months) mean (SD)	12.4 (14.5)	11.2 (9.8)	11.8 (12.6)
Sample collection method^a^			
Urine collection pad	267 (85.6)	219 (86.6)	486 (86.0)
Suprapubic aspiration	35 (11.2)	21 (8.3)	56 (9,9)
Clean catch urine	8 (2.6)	12 (4.7)	20 (3.5)
Catheter	2 (0.6)	1 (0.4)	3 (0.8)
Confirmed UTI	89 (28.5)	54 (21.3)	143 (25.3)
Uropathogens^b^			
*Escherichia coli*	82 (92.1)	43 (79.6)	125 (87.4)
*Klebsiella spp.*	1 (1.1)	5 (9.3)	6 (4.2)
*Enterobacter spp.*	3 (3.4)	4 (7.4)	7 (4.9)
*Enterococcus spp.*	1 (1.1)	–	1 (0.7)
*Proteus spp.*	–	2 (3.7)	2 (1.4)
*Citrobacter spp.*	1 (1.1)	–	1 (0.7)
*Pseudomonas aeruginosa*	1 (1.1)	–	1 (0.7)
Other culture results			
Negative culture	71 (31,8)	54 (27.1)	125 (29.6)
Normal flora	129 (57,8)	113 (56.8)	242 (57.3)
Contaminant growth	23 (10.3)	32 (16.1)	55 (13.0)

*UTI* Urinary tract infection. Infection was confirmed with bacterial culture as golded standard

^a^ Urine collection pad sample was collected from each child. The sample was classified as pad sample if two consecutive samples were collected with urine collection pad

^b^ The bacterial culture findings of confirmed UTI cases

### Urine screening tests

We screened the urine samples using three different methods. First, the diaper-embedded urine test device (Tena-U, commercially available during the study from SCA, Sweden, in 2013–2017 and from Essity Hygiene and Health AB, Sweden, in 2017) was applied to the urine pad within the diaper, as close to the urethral orifice as possible. The device is capable to detect leukocytes and nitrite in the urine sample. Nurses interpreted and recorded the test result (Fig. 1). Second, the urine sample was aspirated from the urine pad, placed under the urine test device for the conventional point-of-care dipstick test (Compur 10-test, Roche Diagnostics GmbH, Switzerland), and interpreted and recorded by the nurses. Third, all aspirated urine samples were analyzed in the laboratory with an automated urine chemistry analyzer (Clinitek Atlas, Siemens Healthcare GmbH, Germany in 2013–2015 and Clinitek Novum in 2016–2017).

### The gold standard for confirming infection

The gold standard for confirming urinary tract infection was quantitative bacterial culture. All children were symptomatic and suspected to have a UTI based on a fever of unknown origin or urinary symptoms. UTI diagnosis was defined as a positive urine bacterial culture with 1) growth of > 10^4^ colony-forming units of the same pathogen per ml in two subsequent clean voided urine or urine collection pad samples; or 2) if only one sample was collected, growth of > 10^5^ colony-forming units of the known uropathogen per ml in the clean voided urine or urine collection pad samples; or 3) any bacterial growth in a urine sample obtained by suprapubic bladder aspiration. Mixed growth, normal flora of the area or any other bacterial growth were considered as contamination and were not defined as UTI. Urine samples were classified as negative if any of the cultures taken from the remained remained negative. The patient records were manually reviewed by pediatric infection specialists and clinical symptoms were evaluated. All the children, whose symptoms did not match with UTI were classified as non-UTI.

### Sample size

During the study period, the pediatric emergency department was estimated to have approximately 30,000 visits. The reported frequency of true UTIs in a population of young children with suspected UTI was estimated to be 25% [13]. We assumed based on our clinical judgement and on a previous meta-analysis [14], that the clinical requirement for sensitivity of leukocytes in screening for culture confirmed UTI was 90%. Because the probability of UTI in the study population was estimated to be 25% and the marginal error was 5%, a sample size of 553 was needed based on the sample size estimation in diagnostic test studies [15].

### Statistical methods

We compared the diagnostic accuracy of the three different urine tests in the screening of bacterial culture confirmed UTIs in children based on 2-way comparisons. For each urine test, the sensitivity (probability that a test result will be positive when the disease is present; i.e., UTI was confirmed by the positive culture), specificity (probability that a test result will be negative when the disease is not present), positive predicted value (PPV; probability that the disease is present when the test is positive), and negative predicted value (NPV; probability that the disease is not present when the test is negative) were calculated for culture-confirmed UTI with a 95% confidence interval. All the comparisons were made for leukocyte and nitrite detection separately. We calculated Cohen’s kappa (κ) in order to estimate agreement between the methods. Statistics were calculated using StatsDirect Statistical Software (version 3.2.8, StatsDirect Ltd., England).

## Results

**Fig. 2:**
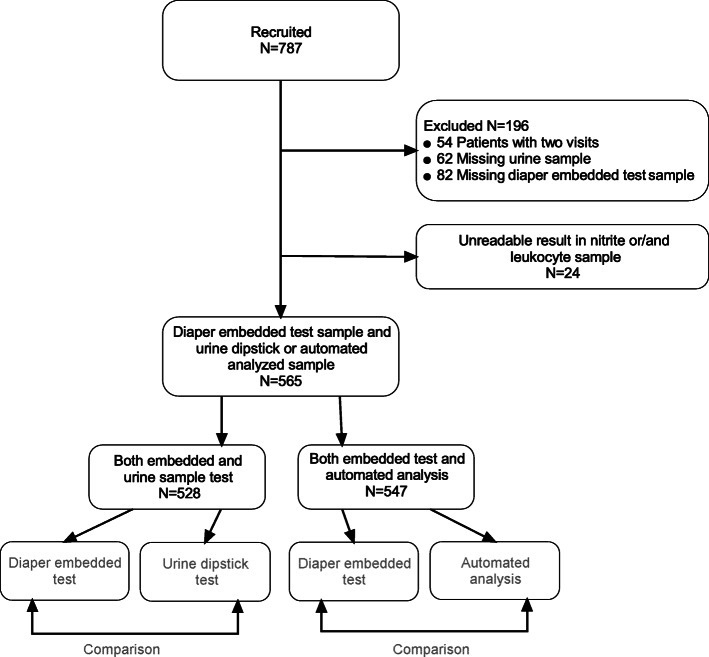
Study flow chart

We recruited 787 children. Altogether, 222 of them were excluded due to earlier participation (*n* = 54), missing study urine sample (*n* = 144), or unclear urine test device result (*n* = 24) (Fig. 2). We used samples of the 565 children with suspected UTI for the comparisons (Fig. 2). The mean age of the children was 11.8 months (SD: 12.6). UTI was confirmed using the bacterial culture (gold standard test) in a total of 143 children, of whom 89 (62.2%) were girls and 54 (37.8%) were boys. The most common uropathogen was *Escherichia coli* in 125 samples (87.4%). Of the remaining 422 samples, culture results were negative for 125 (29.6%) samples, 242 (57.3%) samples were classified as mixed or normal bacterial growth of the area and 55 (13.0%) samples were classified as contaminant growth resulting contamination rate of the positive culture results of 297/565 (52.6%). Suprapubic aspiration was achieved from the 56 children. Of these samples 16 were culture negative and only one (1.8%) was classified as contamination (Table 1).

### Sensitivity and specificity of leukocyte detection

We first compared the diaper-embedded urine test and point-of-care urine dipstick to culture results of the urine samples of 528 children, which were successfully screened using both methods. The sensitivity of the positive leukocyte screening for culture-confirmed UTI was 93.1% for the diaper-embedded urine device and 95.4% for point-of-care urine dipstick test (Table 2). Specificity of leukocytes was 64.4% for the diaper-embedded test and 77.3% for the point-of-care dipstick test. PPV was 46.4% for the diaper-embedded test and 58.1% for the point-of-care dipstick test. NPVs were high for both tests, at 96.6 and 98.1%, respectively (Table 2). Observed agreement between the methods was 81.1% and κ = 0.62 indicated substantial agreement.

**Table 2:** Performance of the diaper-embedded urine test leukocytes in the screening of bacterial culture-confirmed UTI^a^

Leukocytes	Sensitivity	%	(95% CI)	Specificity	%	(95% CI)	PPV	%	(95% CI)	NPV	%	(95% CI)
**Group 1 (*n*** **= 528)**												
Embedded test	122/131	93.1	87.4–96.8	256/397	64.4	59.6–69.2	122/263	46.4	40.2–52.6	256/265	96.6	93.7–98.4
Urine dipstick	125/131	95.4	90.3–98.3	307/397	77.3	72.9–81.4	125/215	58.1	51.2–64.8	307/313	98.1	95.9–99.3
**Group 2 (*n*** **= 547)**												
Embedded test	127/139	91.4	85.4–95.5	260/408	63.7	58.6–68.4	127/275	46.2	40.2–52.3	260/272	95.6	92.4–97.7
Laboratory	123/139	88.5	82.0–93.3	363/408	89.0	85.5–91.8	123/168	73.2	65.9–79.7	363/379	95.8	93.2–97.6

*PPV* Positive predictive value, *NPV* Negative predictive value, *UTI* Urinary tract infection

Group 1: diaper-embedded test vs. urine dipstick test; Group 2: diaper-embedded test vs. laboratory screening test

^a^ UTI was defined as 1) a positive urine culture, defined as growth of > 10^5^ colony-forming units of the same pathogen per ml in two subsequent clean voided urine or urine collection pad samples; 2) growth of > 10^5^ and < 10^4^ colony-forming units of the same pathogen per ml in two subsequent clean voided urine or urine collection pad samples; 3) if only one sample was collected, growth of > 10^5^ colony-forming units of the known uropathogen per ml in the clean voided urine or urine collection pad samples; or 4) any magnitude of bacterial growth in a urine sample obtained by suprapubic bladder aspiration

We then compared urine culture results of 547 samples that were simultaneously screened by the diaper-embedded urine test device and laboratory-based automated urine analysis. In this comparison, sensitivity of the positive leukocyte screening of the diaper-embedded test was 91.4% and that of automated analysis was 88.5% (Table 2). Specificity was 63.7% for the diaper-embedded test device and 89.0% for the automated analysis. PPVs were 46.2% for the embedded tests and 73.2% for the automated analysis, whereas NPVs were 95.6 and 95.8%, respectively (Table 2). Observed agreement between the two methods was 76.0% and κ = 0.52 indicated moderate agreement.

### Sensitivity and specificity of nitrite detection and combined leukocyte and nitrite detection

Sensitivity of the nitrite screening for culture-confirmed UTI in young children was low for the diaper-embedded urine test, urine dipstick test, and automated analysis (Table 3). All tests performed well in terms of specificity (Table 3).

**Table 3:** Performance of the diaper-embedded urine test nitrites in the screening of bacterial culture-confirmed UTI^a^

Nitrite	Sensitivity	%	(95% CI)	Specificity	%	(95% CI)	PPV	%	(95% CI)	NPV	%	(95% CI)
**Group 1 (*n*** **= 528)**												
Embedded test	63/131	48.1	39.3–57.0	384/397	96.7	94.5–98.3	63/76	82.9	72.5–90.1	384/452	85.0	81.3–88.1
Urine dipstick	67/131	51.2	42.3–60.0	379/397	95.5	92.9–97.3	67/85	78.8	68.6–86.9	379/334	85.6	81.9–88.7
**Group 2 (*n*** **= 547)**												
Embedded test	64/139	46.0	37.6–54.7	393/408	96.3	94.0–97.9	64/79	81.0	70.6–89.0	393/468	84.0	80.3–87.2
Laboratory	52/139	37.4	29.4–46.0	401/401	98.3	96.5–99.3	52/59	88.1	77.1–95.1	401/488	82.2	78.5–85.5

*PPV* Positive predictive value, *NPV* Negative predictive value, *UTI* Urinary tract infection

Group 1: diaper-embedded test vs. urine dipstick test; Group 2: diaper-embedded test vs. laboratory screening test

^a^ UTI was defined as 1) a positive urine culture, defined as growth of > 10^5^ colony-forming units of the same pathogen per ml in two subsequent clean voided urine or urine collection pad samples; 2) growth of > 10^5^ and < 10^4^ colony-forming units of the same pathogen per ml in two subsequent clean voided urine or urine collection pad samples; 3) if only one sample was collected, growth of > 10^5^ colony-forming units of the known uropathogen per ml in the clean voided urine or urine collection pad samples; or 4) any magnitude of bacterial growth in a urine sample obtained by suprapubic bladder aspiration

We also calculated sensitivity and specificity for combined leukocyte and nitrite detection for the diaper embedded test. Combining leukocyte and nitrite results for screening slightly increased the sensitivity compared to leukocyte detection alone (95.4% vs 93.1%). However, specificity was lower (63.7% vs. 64.4%).

### Easiness of use, time to the test result and adverse events

The parents found sample collection easy, with a mean visual analog scale value of easiness (ranging from easy = 0 mm to very difficult = 90 mm) of 9.3 mm (SD: 12.3). Of a total of 24 samples (3% of the original 787 samples), the results of the embedded test were unreadable. The nurses found the diaper embedded test device easy to use when compared to conventional testing.

The nurses had the diaper-embedded test results ready immediately after urination was observed. Point-of-care dipstick test, requiring the aspiration of the urine from the pad, was ready in 1–5 min. Laboratory-based automated analysis was received in 30 to 60 min.

No serious adverse events were reported. In two cases, the embedded test adhered with the urine collection pad and damaged the surface of the pad. In both cases, the results were readable and urine samples were successfully aspirated for the bacterial culture. In one case, the diaper-embedded test device was slightly adhered to the skin of the child, leading to mild erythema in the diaper area. Four families (0.7%) reported mild erythema in the child’s diaper area.

## Discussion

In this prospective study, the diaper-embedded urine test device was a sensitive screening method for UTIs in young children. The main clinical benefit of the diaper-embedded urine test device was that the screening test result was immediately available after urination.

Many national and international guidelines recommend non-invasive methods for urine sample collection in children [7]. Most of the European guidelines recommend clean catch methods due to their lower contamination rate compared to other non-invasive methods [16, 17], but the methods are often time consuming and their success rates are often low [4, 18]. Thus, urine pads are still frequently used for urine sample collection in young children. In the present study, the families found immediate urine analysis with the diaper-embedded test device, placed on the urine pad within the diaper, easy and convenient.

According to our results, the sensitivity of the diaper-embedded test device for leukocyte screening was high. Combining nitrite screening with leukocyte screen only slightly increased the sensitivity and resulted in slight decrease in specificity of the tests. In a previous study, urinary leukocyte screening with a conventional dipstick performed well in the UTI diagnostics of febrile infants [9]. Our study shows that the diaper-embedded urine test device performed as well as the conventional dipstick test in the screening of UTIs in young children. For nitrite screening, our results were similar to those of a recent meta-analysis showing the low sensitivity of nitrite sticks in the screening of UTIs in young children [19]. The proportion of contaminated urine samples collected from the diapers was 53%, which is in accordance to previous studies with contamination rates up to 60% [20].

The major strength of our study lies in the prospective setting and large sample size of acutely ill young children with a suspected UTI. Furthermore, different methods were compared using the same urine sample from each child. Finally, UTIs were confirmed using bacterial culture. As a limitation, false positive culture samples affect diagnostics of UTI in young children. Relatively small proportion of the samples were collected with suprapubic aspiration which increases proportion of contaminated samples. However, UTI diagnosis was based on two consecutive samples and clinical symptoms were evaluated from the patient records. This makes probability of false UTI diagnosis smaller. In clinical use price of the diaper-embedded test would be somewhat higher compared to urine dipstick. This could be compensated by saving time of health care professionals as time required to obtain test results is shorter using the diaper-embedded test. However, we did not investigate the cost-effectiveness of the diaper-embedded test device in clinical practice since the present study was designed to evaluate the diagnostic accuracy.

## Conclusions

In conclusion, in this prospective cohort study, diaper-embedded test device appeared to be sensitive and feasible method for screening UTI in acutely ill young children.

## Data Availability

The datasets used and/or analysed during the current study are available from the corresponding author on reasonable request for clinical research purposes.

## Abbreviations

UTI: Urinary Tract Infection
PPV: Positive Predicted Value
NPV: Negative Predicted Value
